# Characteristics of Eye Movements and Correlation to Cognitive Functions in Relation to the Location of Guide Signs and Driving Speed

**DOI:** 10.3390/jemr19020025

**Published:** 2026-03-02

**Authors:** Takaya Maeyama, Hiroki Okada, Daisuke Sawamura

**Affiliations:** 1 Graduate School of Health Sciences, Hokkaido University, Kita 12-jo Nishi 5-chome, Kitaku, Sapporo 060-0812, Hokkaido, Japan; maeyama.takaya.n9@elms.hokudai.ac.jp; 2 Department of Rehabilitation Sciences, Hokkaido University, Kita 12-jo Nishi 5-chome, Kitaku, Sapporo 060-0812, Hokkaido, Japan; d.sawamura@pop.med.hokudai.ac.jp

**Keywords:** driving, eye movement, guide signs, visual recognition, cognitive function, on-board videos

## Abstract

Driving safety critically depends on the ability of drivers to efficiently recognize and process guide sign information under varying traffic conditions. This study examined how driving speed (slow/fast) and guide sign location (front/left) influence eye-movement behavior during guide sign recognition, and how these effects relate to drivers’ cognitive functions and basic demographics. Twenty-four licensed drivers performed a guide sign recognition task using onboard video stimuli, and eye movements based on fixations and saccades were recorded. Generalized linear mixed models with participants as random effects were used to analyze the interactions between driving conditions, cognitive functions, demographics, and eye movement measures. Under low-load conditions, such as slow driving and front-positioned signs, individual differences in cognitive functions, including verbal memory and useful field of view, were strongly reflected in eye-movement behavior. Under high-load conditions characterized by fast driving and left-positioned signs, the influence of cognitive function was reduced, and eye movements were more strongly associated with driving experience. Increasing driving speed was associated with fewer eye movements, whereas the saccade amplitude remained unchanged, indicating the suppression of exploratory eye movements. For left-positioned signs, the fixation duration on the target was maintained, whereas gaze shifts between the forward environment and the sign were reduced.

## 1. Introduction

Driving is an essential part of daily life in modern society, contributing significantly to both activities of daily living and quality of life [1,2], and serves as a vital means of transportation that promotes individual social participation [1,3]. However, a decline in visual attention and information processing ability while driving significantly increases the risk of serious traffic accidents [4,5,6]. In particular, safe driving requires not only accurately perceiving and responding to the forward environment, such as pedestrians, other vehicles, and oncoming traffic, but also correctly recognizing and processing information from traffic and guide signs [7,8].

Information processing while driving primarily relies on visual search, and efficient, well-structured eye movements have been reported to be strongly associated with hazard recognition ability [9,10]. Previous studies on the recognition of textual information on guide signs have shown that factors such as the amount of information presented [11], arrangement of place names [11,12], and font size [13] affect gaze behavior. However, these studies have not sufficiently examined the relationship between fundamental abilities—such as cognitive function and driving experience—and the recognition of guide signs. Moreover, the visibility of guide signs placed outside the driver’s forward field-of-view has not been adequately investigated. Thus, this study aimed to examine eye movements during guide sign recognition from two distinct analytical perspectives.

The first perspective treats the two driving condition factors—driving speed (slow vs. fast) and sign location (front vs. left)—independently, and aims to clarify, through comparisons between slow and fast speeds and between front and left placements, how each factor alone influences eye movements and how driver characteristics, such as cognitive functions and driving experience, are involved in modulating these effects.

The second perspective focused on comparing the characteristics of eye movements across four driving conditions: slow–front, slow–left, fast–front, and fast–left. This analysis aims to clarify the extent to which visual search is constrained and the types of search strategies that emerge for each specific situation encountered in real driving.

In this study, we examined how the contributions of cognitive functions and driver characteristics to eye-movement behavior during guide sign recognition changed at different driving speeds and sign locations. Hypothesis 1 posited that the relationships between eye movements and cognitive functions, as well as driver characteristics, differ depending on driving speed (slow vs. fast) and sign location (front vs. left). Hypothesis 2 assumed that, as driving speed increases, the number of fixations and saccades during guide sign recognition decreases. Furthermore, Hypothesis 3 predicted that when guide signs are placed on the left side, eye-movement patterns differ from those observed under front placement, with particular constraints on exploratory eye movements that alternate between the guide sign and the forward-driving environment.

This study is an exploratory investigation of eye-movement behavior during guide sign recognition in healthy adults, examining both individual factors, such as cognitive function and driver characteristics, and driving condition factors, namely, driving speed and sign location. By combining analyses that independently compare driving speed (slow vs. fast) and sign location (front vs. left) with comparisons across conditions that integrate both factors, this study aimed to provide a multifaceted clarification of which factors constrain eye-movement behavior and the driving situations under which these constraints arise. These findings elucidate visual search during driving and serve as a foundational resource for future guide sign designs and safe driving support systems.

## 2. Materials and Methods

### 2.1. Participants

The sample size was determined using G*Power version 3.1.9.7 [14]. Considering this study as an exploratory investigation of the relationship between eye-movement behavior during guide sign recognition and cognitive functions, a medium-to-large effect size was assumed for simple correlations (|ρ| = 0.50). With the significance level set at α = 0.05 (two-tailed) and statistical power at 1 − β = 0.80, the theoretically required minimum sample size was 29 participants.

In this study, 24 participants were included, and each participant provided eye-movement data under multiple conditions, combining driving speed (slow vs. fast) and sign location (front vs. left). Generalized linear mixed-effects models (GLMMs) were employed for statistical analysis, with participants treated as random effects, allowing multiple observations obtained from the same participant to be analyzed within a hierarchical structure. GLMMs enable the simultaneous estimation of the effects of driving conditions and cognitive functions or basic attributes, while accounting for inter-individual variability, making them an appropriate analytical approach for the data structure of this study [15,16].

In addition, sensitivity analysis indicated that with a sample size of n = 24, effect sizes of approximately |ρ| = 0.55 or greater would be detectable in simple correlation analyses. This study was designed as an exploratory investigation aimed at elucidating the relationships between cognitive functions and eye-movement behavior during guide sign recognition, an area that has not been sufficiently examined to date, as well as the effects of driving condition factors, such as driving speed and sign location. Accordingly, the study design emphasized the identification of substantively meaningful effects rather than the exhaustive detection of small effects [17].

The participants in this study were local residents of a major city in Japan. Recruitment was conducted primarily at medical institutions and universities in the medical sciences, using flyers and posters. The inclusion criteria were as follows: (1) 20–59 years of age with a valid driver’s license, (2) corrected or uncorrected visual acuity of 0.6 (decimal notation; equivalent to logMAR 0.22) or better, (3) no history of neuropsychological conditions, (4) no history of epilepsy, and (5) no history of ophthalmologic diseases, such as cataracts or glaucoma.

Finally, 24 participants (8 males and 16 females) were included in the study. The mean age, mean driving history, and mean education were 36.5 ± 12.8 (21–59), 14.8 ± 14.4 (0–40), and 15.0 ± 1.9 (12–20) years, respectively. The sample exhibited a sex imbalance with more female participants (n = 16) than male participants (n = 8). However, this study was not designed to examine sex differences; rather, it was an exploratory investigation focusing on the relationships between eye-movement behavior during guide sign recognition and cognitive functions under different driving conditions. Therefore, the potential impact of this sex imbalance on the interpretation of the main findings is limited.

### 2.2. Apparatus

To collect data using on-board videos, a stimulus presentation monitor (iiyama ProLite B2480HS, Mouse Computer Co., Ltd., Tokyo, Japan) was used. The monitor measured 29.5 cm in height and 52.35 cm in width, with a screen resolution of 1920 × 1080 pixels and a brightness of 300 cd/m^2^. The viewing distance was set to 57 cm, corresponding to a visual angle of approximately 49.4° horizontally and 29.1° vertically. This range was considered sufficient for participants to shift their gaze and focus on visual information within the on-board video stimuli [18], and it was unlikely that gaze behavior was restricted by the size of the monitor.

Participants’ eye movements were recorded using an eye tracker (Tobii X60 Eye Tracker; Tobii, Danderyd Municipality, Sweden) with a sampling rate of 60 Hz. Tobii Studio version 3.1.6 (Tobii) was used to present the stimuli and analyze the eye-movement data collected by the eye tracker.

### 2.3. Stimuli

The on-board videos used for data collection were recorded under the following four conditions: (1) slow speed (40–60 km/h) with guide signs located ahead and in the lane (slow–front condition), (2) slow speed with guide signs located on the left side (slow–left condition), (3) fast speed (100–120 km/h) with guide signs located ahead and in the lane (fast–front condition), and (4) fast speed with guide signs located on the left side (fast–left condition). Ten videos were recorded for each condition. Videos under the slow-speed conditions were filmed on general roads, while those under the fast-speed conditions were filmed on highways.

The guide sign videos used in this study were all recorded from the driver’s perspective (first-person view) using on-board cameras. External information such as navigation voice guidance and subtitles was removed to reproduce natural visual conditions closely resembling real driving environments. In all videos, the visible duration of the guide signs was uniformly set to 4 s, ensuring no differences in presentation time across conditions. This 4 s duration was based on the average fixation times required for traffic sign recognition reported in previous studies [19,20] and is considered sufficient for perceiving and processing sign information.

The reason for selecting the speed range of 40–60 km/h for the (1) and (2) slow-speed conditions was, first, that a previous study on brain activity during driving on Japanese roads [21] used 40 km/h, and the driving environment in this study was similar to that. Second, 60 km/h is the legal speed limit on general roads in Japan [22]. Additionally, based on findings that drivers on Japanese highways generally travel at speeds of 100–120 km/h [23], the (3) and (4) fast-speed conditions were defined as 100–120 km/h.

The average driving speeds for the slow–front, slow–left, fast–front, and fast–left conditions were 51.1 km/h, 49.9 km/h, 103.1 km/h, and 101.3 km/h, respectively, all within the designated ranges. The experimental conditions comprised two driving speeds (Slow and Fast) and two sign locations (Front and Left), resulting in four combinations. These factors were later modeled as fixed effects in generalized linear mixed models (GLMMs) to examine their main effects and interactions with eye movement measures. To confirm that these minor differences in average speed did not systematically vary by sign location, we additionally examined the mean driving speed using a generalized linear mixed-effects model with driving speed (slow vs. fast) and sign location (front vs. left) as fixed effects and participants and videos as random intercepts, which revealed no significant main effect of sign location or speed × location interaction. Furthermore, to eliminate the effects of acceleration, deceleration, and lateral steering, only segments with acceleration below 0.1 G were used to measure gaze behavior during stable driving.

Regarding the placement of guide signs, two categories were defined: (1) and (3) were front conditions, and (2) and (4) were left conditions. The front condition refers to signs located directly ahead in the direction of the vehicle’s travel, installed within the driving lane. In contrast, the left condition refers to signs installed to the left side of the driving lane. On multi-lane roads, this includes signs located to the left of the lane in which the vehicle is traveling. Even when the vehicle is traveling in the leftmost lane or on a single-lane road, signs placed near sidewalks or side roads were classified as left condition.

The guide signs displayed destination names (such as city/town names, interchange names, and junction names) that are actually installed on general roads and highways. The selected destination names were chosen from locations geographically unfamiliar to the participants (areas distant from their homes) to eliminate the influence of prior knowledge. The number of destination names per sign ranged from one to eight. A Kruskal–Wallis test with Bonferroni correction was conducted to compare variations in the number of destination names across the four conditions (speed × sign location), and no significant differences were found.

The display positions of each destination name on the guide signs were classified into five relative regions (top, bottom, left, right, center) based on the visual layout of the sign, with the screen center as a reference point. The classification criteria were determined using a grid that divided the entire rectangular area of the sign into thirds vertically and horizontally, assigning each destination name to the region containing its primary centroid coordinates. To control for potential spatial biases in visual attention, the stimuli were designed such that destination names were evenly distributed across the five regions (two names per region, totaling 10 locations).

**Figure 1:**
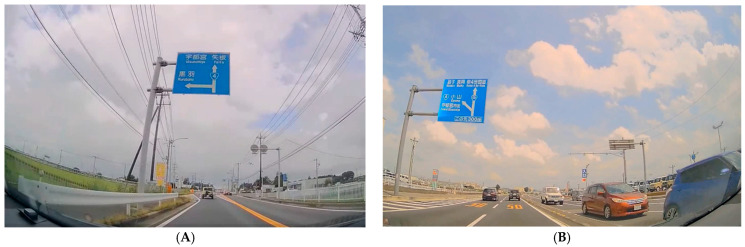
Example of placement of a guide sign in on-board videos. (**A**) front condition; (**B**) left condition. The guide signs contain Japanese place names (shown in both Japanese characters and Romanized form), such as *Utsunomiya*, *Kurobane*, and *Yaita* in (**A**), and *Koyama*, *Maoka*, and *Center Utsunomiya* in (**B**), reflecting actual road signs used in Japan.

It should be noted that in this study, the “display position of destination names” was not treated as a primary variable for evaluating relationships with eye movements or cognitive function and was not included in the statistical analyses. Examples of sign layouts are shown in Figure 1A,B.

The font size of the destination names on the guide signs was set according to Japanese regulations based on road sign design standards. Character heights of 20–30 cm were used for the slow-speed conditions, and 50 cm for the fast-speed conditions.

### 2.4. Definition of Visual Recognition

The visual recognition assessment in this study was based on previous research on the visual recognition of obstacles [24,25] and road signs [26,27,28]. The following four eye-movement metrics were used as indicators: fixation duration (ms), number of fixations, saccade amplitude (°), and number of saccades.

Fixation duration and number of fixations have been widely used in previous studies on road signs [12,29] and are considered valid evaluation metrics in this study as well.

Fixation duration is defined as the average fixation time on destination names within the guide signs [30]. Longer fixation durations are interpreted as indicating greater allocation of attention to the target [28,31].

The number of fixations refers to the total count of fixations made when recognizing destination names and reflects the distribution of visual attention [26,32]. Generally, a higher number of fixations is considered indicative of increased visual attention.

Additionally, based on previous research showing that Japanese readers process approximately five characters within a visual angle of 5° [33,34], this study regarded gaze points within 5° of visual angle from a destination name as a fixation.

Saccade amplitude refers to the average amplitude of saccadic eye movements directed toward destination names within the guide signs. Larger saccade amplitudes suggest that the destination was clearly recognized and that gaze shifts were more direct and efficient [35,36]. Therefore, saccade amplitude can be interpreted as an indicator of the difficulty of destination recognition.

The number of saccades indicates the total count of gaze shifts directed toward destinations within the guide signs. A lower number of saccades may suggest more efficient visual search behavior [26,28]. In this study, saccades were defined as gaze shifts either from fixation points outside the sign to destination names within the sign, or between destination names within the sign [37].

**Figure 2:**
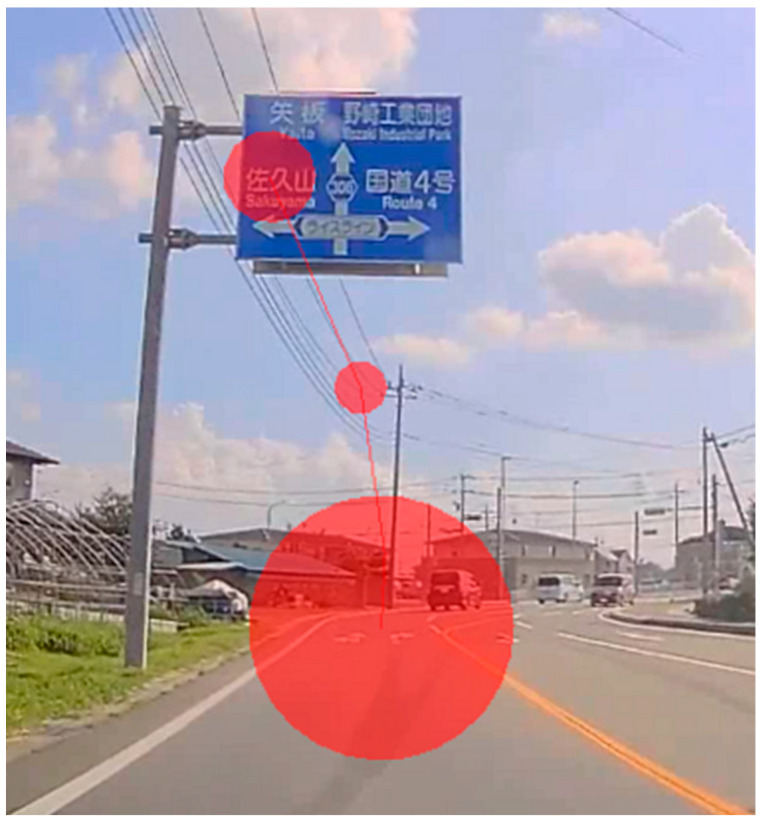
Representative gaze plot showing fixations (red circles) and saccades (connecting lines) during the recognition of destinations on guide signs. This example illustrates the gaze trajectory when participants were instructed to recognize the destination name “佐久山” (*Sakuyama*, a Japanese place name displayed on the actual road sign). Although dynamic heat maps could not be generated in Tobii Studio version 3.1.6, these gaze plots confirm that participants visually attended to the guide sign during the task. The highlighted areas are overlaid to illustrate gaze distribution and do not affect the scientific interpretation of the figure.

Measurement of these eye movement metrics was conducted using the Tobii Studio’s I-VT fixation filter (Tobii) [38]. Fixations were defined as those lasting at least 60 ms with an angular difference of <1° between two consecutive fixations, and saccades were defined as eye movements with velocities of 30°/s or higher. Analyses were performed using the average values from both eyes. Figure 2 shows an example of the recorded gaze trajectories (fixations and saccades) obtained using the Tobii X60 eye tracker while the participants recognized the destination name on the guide sign. This figure illustrates how the gaze behavior was captured and analyzed in this study.

In this study, each metric was evaluated based on raw data without normalization (e.g., fixation count or average fixation duration) in order to preserve natural gaze behavior, cognitive load, and individual differences in visual search strategies. This approach is considered effective for exploratory studies involving road signs and driving-related visual tasks [27,29], and is suitable for capturing variations in cognitive flexibility and adaptive gaze behavior.

Furthermore, the use of raw data is expected to facilitate future comparisons with normalized metrics and to support the visualization of strategy differences according to driver characteristics.

### 2.5. Assessment: Cognitive Functions

To assess the cognitive functions necessary for driving, based on previous studies [39,40,41], the Trail Making Test—Japanese Edition (TMT-J) A and B [42] was used to assess the attention and processing speed required for driving [43,44]. The TMT is a widely used test that assesses processing speed, attention, and working memory, which are essential for driving [40,43,44,45]. The Wechsler Memory Scale—Revised (WMS-R) [46] was used to evaluate verbal and visual memory. The Zoo Map Test (ZMT) of the Behavioral Assessment of Dysexecutive Syndrome (BADS) [47] was used to assess planning. The ZMT has been reported to be significantly related to the assessment of driving skills [48,49]. TMT-J A and B were measured in time (s), and the WMS-R and BADS scores were calculated.

To assess the useful field of view (UFOV), we also performed the Double Decision test within BrainHQ^®^ [50], following previous studies [51,52,53,54]. The assessment consisted of two different cars, one in the center of the screen and a road sign (Route 66) somewhere in the peripheral area, which appeared simultaneously for a limited time, along with the disturbing elements, and then disappeared immediately, followed by the type of car and the position where the road sign appeared.

The results of the cognitive function assessments described above are presented in Table 1. These assessments were conducted prior to main data collection.

### 2.6. Data Collection: Procedures

Data collection was conducted in a quiet room with no external light. A five-point calibration was performed for each participant. Based on a previous study [26], participants were instructed to imagine that they were actually driving toward a destination. This was intended to elicit natural attention allocation and eye movements, not just passive observation, simulating the perspective of a driver behind the wheel.

During data collection, participants sat 57 cm from the monitor and viewed on-board videos under four conditions (driving speed × sign position).

The sequence of video presentation was as follows: first, the destination name was displayed for 7 s; next, participants fixated on a central cross for 3 s; finally, an on-board video with a guide sign was shown for 7 s. Calibration was performed before each condition trial. The presentation order was fixed from the slow-speed condition (general roads) to the fast-speed condition (highways), prioritizing natural progression in real driving over counterbalancing. To minimize learning effects due to fixed order, the position of the destination name on each guide sign was systematically varied among upper, lower, left, right, and center positions, ensuring that the same position was not repeated consecutively.

After each video, participants were asked whether they had correctly recognized the destination name, and all reported that they had. This served as a basic performance check to ensure the validity of the eye movement data.

**Figure 3:**
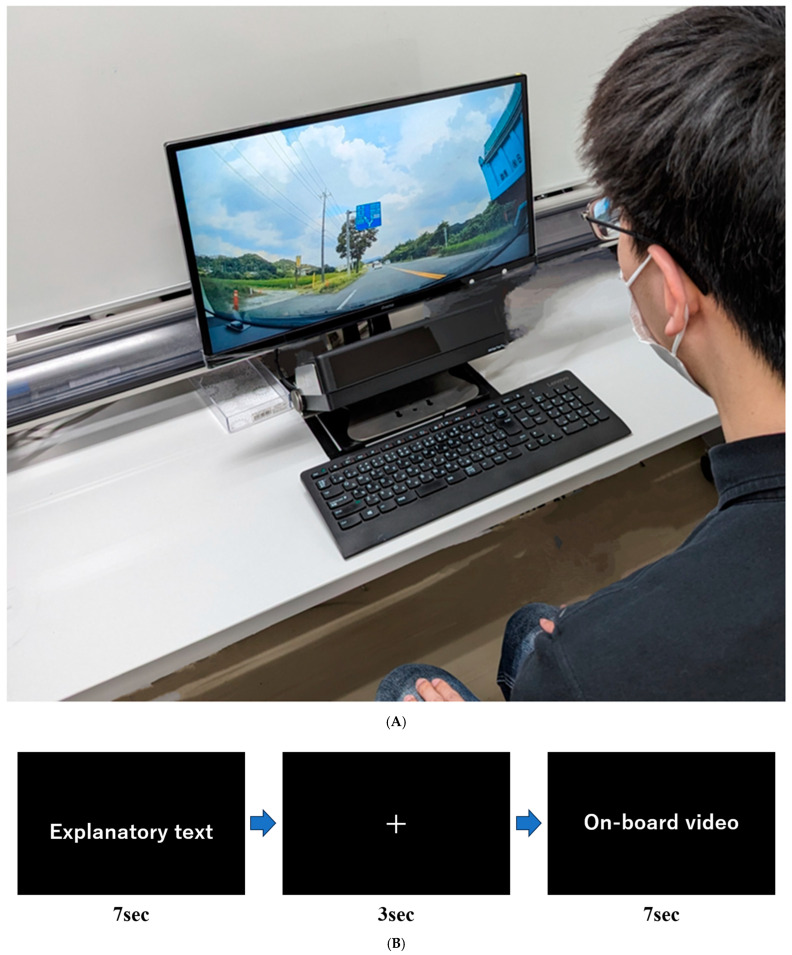

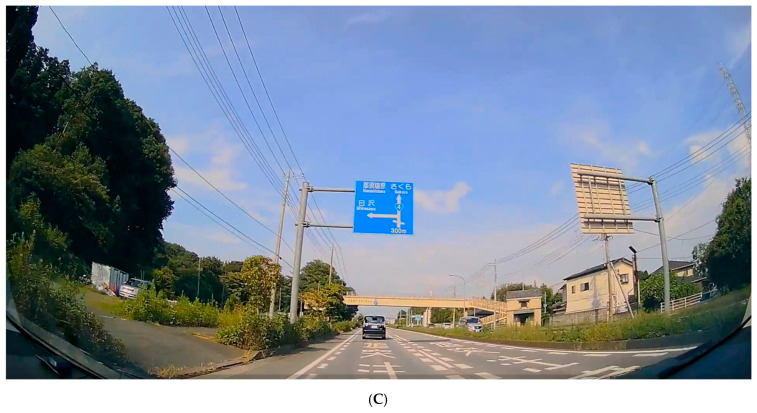
Data collection setting and sequence of data collection. (**A**) Setting of the data collection. (**B**) Schematic of the data collection flow. This sequence is repeated 10 times in each video condition. (**C**) A screenshot taken from the actual on-board video used in this study. The guide sign includes Japanese place names (e.g., *Shirasawa*, *Nasu-Kogen*, and *Sakura*), presented in both Japanese characters and Romanized form, as they appear on real road signs in Japan.

Figure 3A–C show an overview of the setup, data collection procedure, and an example of the video stimuli.

### 2.7. Statistical Analysis

Statistical analyses were conducted to examine the relationships between eye movement measures during guide sign recognition and participant characteristics (cognitive functions and basic demographics) (Analysis 1) and to investigate the effects of driving conditions (driving speed and sign location) on eye-movement behavior (Analysis 2).

All eye movement measures (fixation duration, number of fixations, saccade amplitude, and number of saccades) were analyzed using GLMMs. In each model, an appropriate link function *g*(*·*) was specified according to the scale properties of the dependent variable, and a normal distribution was assumed for all eye movement measures in the present study. The linear predictor included covariates (cognitive functions and basic demographics), driving conditions, driving speed (slow/fast), guide sign location (front/left), and their interaction terms as fixed effects. To account for the dependence of repeated measurements obtained from the same participant and inter-individual variability, participants were included in the models as random intercepts (1|*ID*), where ID denotes each participant.

In the GLMM analyses, simple slope tests were conducted only when the interaction effects were statistically significant and at each condition level, the relationships between covariates and eye movement measures (Analysis 1). For Analysis 2, when a significant interaction between driving speed and sign location was observed, post hoc pairwise comparisons among the four driving conditions were conducted with multiple-comparison correction. When no interaction was observed, only the main effects were interpreted. In addition, to appropriately estimate the interaction effects, the model convergence and stability of parameter estimates were examined, and random slopes accounting for individual differences in interaction-related effects were included when necessary. For fixed effects, estimated coefficients (β), t values, *p* values, effect sizes (η^2^), and 95% confidence intervals (95% CIs) were reported.

#### 2.7.1. Preprocessing: Relationships Among Cognitive Functions and Demographic Variables

Before conducting the GLMM analyses, potential multicollinearity was examined by calculating Pearson’s correlation coefficients between the basic attributes and cognitive function test scores. Several moderate correlations were observed within the same cognitive domains, such as between TMT-J A and B (r = 0.56, *p* < 0.01), between BADS ZMT and UFOV (r = 0.49, *p* = 0.01), and between TMT-J B and WMS-R Visual Memory (r = −0.44, *p* = 0.03). Age and driving history also showed moderate relationships with cognitive measures. A complete correlation matrix is provided in the Supplementary Materials, Table S1 and Figure S1. To avoid multicollinearity, each covariate was entered separately into the GLMM analyses.

#### 2.7.2. Analysis 1: Effects of Driving Speed and Guide Sign Location on Associations Between Eye Movements and Driver Characteristics

In Analysis 1, two driving condition factors—driving speed (slow vs. fast) and guide sign location (front vs. left)—were treated independently to examine how differences in each condition alter the associations between eye-movement behavior and cognitive functions or basic driver demographics. This analysis did not primarily focus on the main effects of the driving conditions themselves but on the interactions between each driving condition and covariates (cognitive functions and basic demographics) to identify the driver characteristics that contribute to eye-movement behavior under different conditions.

Based on the preprocessing results, covariates (*C*) were entered into the models individually. To examine the interactions between each covariate and the driving conditions, driving speed (slow/fast) and sign location (front/left) GLMMs were employed. When significant interactions were detected to clarify condition-specific associations. Accordingly, when the interaction term was significant, the relationships between each covariate and eye movement measure were evaluated separately at each condition level (slow vs. fast; front vs. left).

Two models are constructed for each covariate. Although the fixed-effects structure was identical across models, it differed in the driving conditions included in the interaction term. Therefore, the two models are interpreted independently.

Model 1A: location × covariate interaction model (GLMM)

$$g(E[Y_{ij}])=\beta_{0}+\beta_{1}{\mathrm{Speed}}_{ij}+\beta_{2}{\mathrm{Location}}_{ij}+\beta_{3}C_{i}+\beta_{4}(\mathrm{Speed}\times \mathrm{Location}{)}_{ij}+\beta_{5}(\mathrm{Location}\times C{)}_{ij}+(1|ID).$$

Model 1B: speed × covariate interaction model (GLMM)

$$g(E[Y_{ij}])=\beta_{0}+\beta_{1}{\mathrm{Speed}}_{ij}+\beta_{2}{\mathrm{Location}}_{ij}+\beta_{3}C_{i}+\beta_{4}(\mathrm{Speed}\times \mathrm{Location}{)}_{ij}+\beta_{5}(\mathrm{Speed}\times C{)}_{ij}+(1|ID).$$

Here, $Y_{ij}$ represents the eye movement measure exhibited by participant i in trial j. $\beta_{0}$ denotes the intercept; $\beta_{1}$ and $\beta_{2}$ represent the main effects of driving speed and sign location, respectively; and $\beta_{3}$ indicates the association between the covariate and the eye movement measure under the reference condition. $\beta_{4}$ represents the interaction between driving speed and sign location. The parameter of primary interest in this study, $\beta_{5}$, represents the interaction between the covariate and the driving condition—specifically, Speed × *C* in Model 1A and Location × *C* in Model 1B. When $\beta_{5}$ was statistically significant, simple slopes were calculated for each condition level to examine under which conditions the association between the covariate and the eye movement measure was significant.

#### 2.7.3. Analysis 2: Comparison of Eye Movements Across Driving Conditions

In Analysis 2, situations in which driving speed and sign location were operated simultaneously were assumed, and eye-movement behavior itself was compared across four driving conditions: slow–front, slow–left, fast–front, and fast–left. This analysis did not aim to examine associations with specific covariates but to clarify the driving conditions under which visual search is more constrained.

Accordingly, models were constructed that included the main effects of driving speed and sign location as well as their interaction (speed × location). The analysis procedure was first tested for the presence of an interaction effect; subsequently, the effects of the main factors (driving speed and sign location) were evaluated.

Model 2A/model 2B

$$g(E[Y_{ij}])=\beta_{0}+\beta_{1}{\mathrm{Speed}}_{ij}+\beta_{2}{\mathrm{Location}}_{ij}+\beta_{3}(\mathrm{Speed}\times \mathrm{Location}{)}_{ij}+(1|ID).$$

The model structure and notation definitions are the same as those in Analysis 1.

Statistical analyses were conducted using JMP^®^ 19 Student Edition (SAS Institute Inc., Cary, NC, USA). The significance level was set at *p* < 0.05.

## 3. Results

### 3.1. Results from Analysis 1: Interaction Effects Between Driving Conditions and Driver Characteristics

#### 3.1.1. Interaction Effects of Driving Speed (Slow/Fast) and Cognitive Functions/Basic Demographics on Eye Movement Measures

In this study, multiple interactions between driving speed and covariates were observed for the number of fixations and saccades. The results of the interactions between driving speed and cognitive function as well as basic demographics across the four eye movement measures are presented in Table 2.

**Figure 4:**
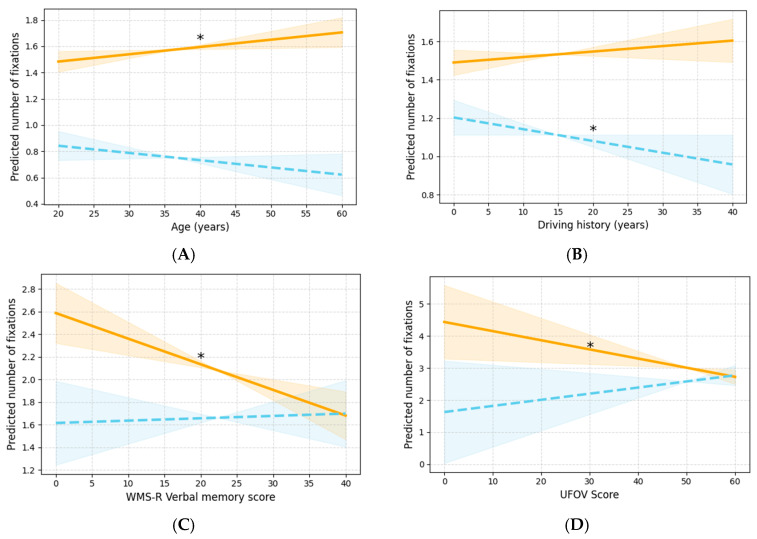
Graphs illustrating the interactions between each covariate and driving speed for the number of fixations. The orange solid lines represent the slow condition, and the blue dashed lines represent the fast condition. Graphs marked with an asterisk indicate significant effects, and the shaded areas around the lines denote the 95% CIs. (**A**) Interaction with age, (**B**) interaction with driving history, (**C**) interaction with WMS-R verbal memory, and (**D**) interaction with UFOV. CI, confidence interval; UFOV, useful field of view; WMS-R, Wechsler Memory Scale—Revised.

Regarding the number of fixations, significant interactions with driving speed were observed for age, driving history, WMS-R verbal memory, and UFOV. Age and the two cognitive function measures showed significant slopes only in the slow condition, whereas driving history exhibited a significant slope in the fast condition (Figure 4A–D).

**Figure 5:**
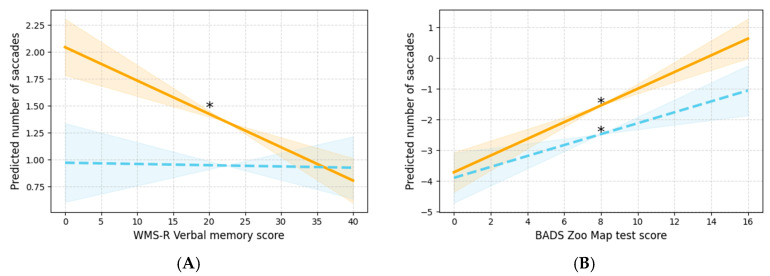
Graphs illustrating the interactions between each covariate and driving speed for the number of saccades. The orange solid lines represent the slow condition, and the blue dashed lines represent the fast condition. Graphs marked with an asterisk indicate significant effects, and the shaded areas around the lines denote the 95% CIs. (**A**) Interaction with WMS-R verbal memory and (**B**) interaction with ZMT. ZMT, Zoo Map Test; CI, confidence interval; WMS-R, Wechsler Memory Scale—Revised.

Regarding the number of saccades, significant interactions with driving speed were observed for WMS-R verbal memory and ZMT. For the WMS-R verbal memory, the slope was significant only under slow conditions, whereas for the ZMT, significant slopes were observed under both slow and fast conditions (Figure 5A,B).

Regarding the number of fixations, a higher age was associated with higher values of the eye movement measure under the slow condition. By contrast, for driving experience under the fast condition and for WMS-R and UFOV under the slow condition, higher experience or higher scores were associated with lower values.

Higher WMS-R verbal memory scores were associated with lower values for the number of saccades, whereas higher ZMT scores were associated with higher values for the eye movement measure.

In the driving speed model, no interaction effects were observed for fixation duration or saccade amplitude.

#### 3.1.2. Interaction Effects of Guide Sign Location (Front/Left) and Cognitive Functions/Basic Demographics on Eye Movement Measures

In this study, multiple interactions between sign location and covariates were observed for the number of fixations and saccades. The results of the interactions between sign location and basic demographics, as well as cognitive functions across the four eye movement measures, are presented in Table 3.

**Figure 6:**
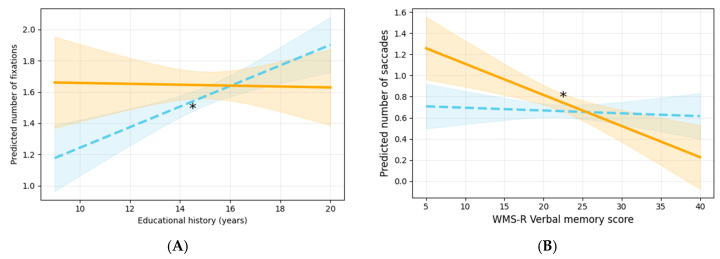
Graphs illustrating the interactions between sign location and each covariate. The orange solid lines represent the front condition, and the blue dashed lines represent the left condition. Graphs marked with an asterisk indicate significant effects, and the shaded areas around the lines denote the 95% confidence intervals. (**A**) Interaction between educational history and the number of fixations, and (**B**) interaction between WMS-R verbal memory and the number of saccades. WMS-R, Wechsler Memory Scale—Revised.

First, regarding the number of fixations, the interaction with educational history was statistically significant. Specifically, the slope for educational history was significant on the left. Regarding the number of saccades, the interaction with the WMS-R verbal memory was significant, with a significant slope observed in the front condition (Figure 6A,B).

In the left condition, a higher educational history was associated with a greater number of eye movements. Additionally, in the front condition, higher WMS scores were associated with fewer saccades.

No interaction effects were observed for fixation duration or saccade amplitude in the sign location model.

### 3.2. Results from Analysis 2: Comparison of Eye Movements Across Driving Conditions: Main Effects of Driving Speed and Guide Sign Location on Eye Movement Measures

Because the interaction effects were not significant for any of the four measures, the main effects of driving speed (slow/fast) and sign location (front/left) were tested. Table 4 presents the results of the study.

For fixation duration and number of fixations, only the main effect of driving speed was statistically significant. Regarding the number of saccades, the main effects of both driving speed and sign location were significant. As the driving speed increased, fixation duration, number of fixations, and number of saccades decreased.

No main effect of sign location was observed for measures other than the number of saccades. For saccade amplitude, neither driving speed nor sign location had a significant effect.

**Figure 7:**
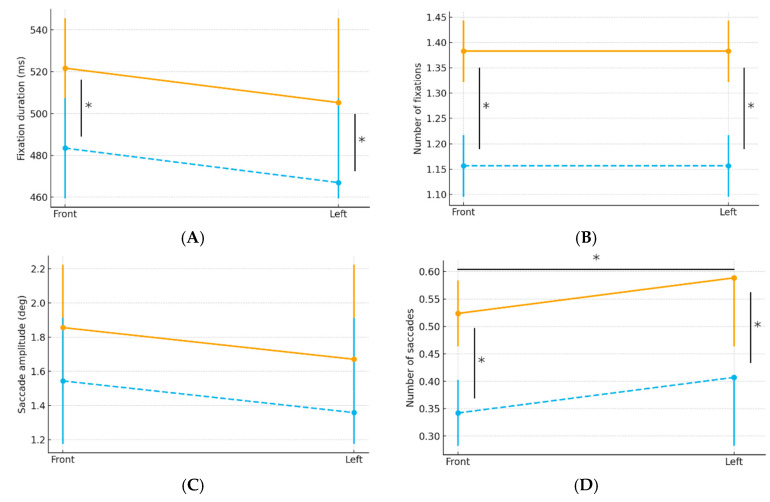
Comparison of eye movement measures across driving conditions. The orange solid lines represent the slow condition, and the blue dashed lines represent the fast condition. Error bars indicate 95% CIs. Asterisks denote conditions with statistically significant differences. (**A**) Fixation duration, (**B**) number of fixations, (**C**) saccade amplitude, and (**D**) number of saccades. CI, confidence interval.

Graphs illustrating the comparison results are shown in Figure 7A–D.

## 4. Discussion

### 4.1. Summary of Key Findings

This study examined the relationship between eye-movement behavior and cognitive functions during guide-sign recognition while driving, using actual onboard videos. The two main findings are that (1) the driver characteristics relied upon during the guide sign recognition shift depend on the level of task demand and (2) driving speed and sign location intensify constraints on eye-movement behavior.

### 4.2. Cognitive Functions Dominate Eye-Movement Behavior Under Low Task Demands, Whereas Driving Experience Predominates Under High Task Demands

The covariate analyses revealed multiple interactions with driving speed. For the number of fixations, interactions were observed with age, driving history, verbal memory, and UFOV, whereas interactions with verbal memory and planning ability were observed for the number of saccades. Thus, contrary to the initial assumption of a simple relationship in which both cognitive functions and driving experience become more important as speed increases, the amount of leeway available for visual search varies depending on the driving conditions. In turn, the individual characteristics that contribute to eye-movement behavior may change according to the degree of leeway.

Specifically, under the slow condition, relatively greater temporal flexibility for visual search allowed individual differences in cognitive functions such as verbal memory and UFOV to be more readily reflected in eye-movement behavior. Under slow conditions, the number of fixations increased with age, whereas drivers with higher verbal memory and UFOV scores exhibited fewer fixations. This pattern indicates the coexistence of two strategies: one in which search volume is increased to compensate for age-related declines in processing speed [55] and another more efficient strategy in which search volume is reduced by relying on internal representations and a broader useful field of view. Thus, under low-demand conditions, differences in “how information is processed,” that is, cognitive functions, are more likely to manifest as differences in eye-movement behavior.

By contrast, in the fast condition, none of the cognitive function measures showed significant associations with eye-movement behavior, whereas driving history alone exhibited a negative association with the number of fixations. During fast-speed driving, the demands for vehicle control and forward monitoring increase [56], substantially constraining the freedom available for a visual search. Consequently, automated search strategies shaped by accumulated experience may be more effective than flexible cognitive adjustments. Therefore, under high-demand conditions, eye-movement behavior may be governed by driving experience rather than cognitive functions.

A similar pattern of demand-dependent adjustment in eye-movement behavior was observed for interactions involving sign locations. In the frontal condition, verbal memory was associated with the number of saccades, suggesting that, when information is presented within the forward visual field and perceptual demands are relatively low, efficient information processing based on verbal internal representations is possible. Thus, drivers with a greater capacity to temporarily maintain and process sign information may have suppressed exploratory eye movements that alternate between the sign and surrounding environment, resulting in more stable eye-movement behavior.

By contrast, in the left condition, educational history was associated with the number of fixations. This finding can be interpreted as reflecting individual differences in the selection of search strategies in situations with substantial attentional allocation constraints, where drivers must process lateral information while maintaining a forward gaze. Recognizing left-side guide signs is not merely a matter of insufficient processing time, but rather a task that requires strategic adjustment of how the gaze is allocated between the forward environment and the sign [57]. Higher educational attainment may reflect greater sophistication in strategy selection and structuring of searches under constrained gaze allocation, such that drivers with a higher educational history adopted a strategy of increasing the number of fixations to avoid missing critical information.

Overall, in high-demand situations where freedom of exploration is constrained by sign location, eye-movement behavior is generally adjusted based more on driving experience and strategic adaptation than on immediate cognitive processing capacity. Conversely, under lower-demand conditions such as front sign placement, cognitive functions, particularly verbal memory, play a primary role in shaping eye-movement behavior. This pattern suggests a structural shift, in which cognitive functions dominate under low-demand conditions, whereas driving experience and search strategies become the principal determinants under high-demand conditions.

Even under low-demand conditions, different types of cognitive functions are reflected in eye-movement behavior in distinct ways. In the present study, higher WMS and UFOV scores were associated with fewer fixations and saccades, whereas higher scores on the ZMT, which assesses planning ability, were associated with a greater number of saccades. WMS and UFOV reflect the ability to internally maintain and integrate sign information, thereby reducing the need for external exploration, which likely manifests as reduced search volume [58]. By contrast, ZMT reflects the ability to structure the order and allocation of search under constraints, and the increase in saccades may reflect active gaze behavior, in which information is constructed through repeated alternations between the sign and the surrounding environment [59]. In this study, ZMT was also significantly associated with the number of saccades under the fast condition, suggesting that even in high-demand situations with limited temporal and attentional resources, planning ability may contribute by enabling drivers to maintain and adapt their visual search within a restricted range, rather than abandoning exploration altogether. In summary, although a broad range of cognitive function differences are reflected in eye-movement behavior under low-demand conditions, many of these functions become less effective under high-demand conditions, leaving only functions such as planning ability, which regulates the structure of exploration, partially operative, indicating a stepwise shift in the determinants of eye-movement behavior.

### 4.3. Increased Driving Speed and Left-Side Sign Placement Are Conditions That Intensify Constraints on Eye-Movement Behavior

An analysis of eye-movement behavior showed that as driving speed increased, fixation duration, number of fixations, and number of saccades decreased. Thus, higher speeds constrain the time available for processing visual information [27,60,61], thereby suppressing the overall visual search behavior. Importantly, no changes in saccade amplitudes were observed as a function of speed or sign location. Drivers did not compensate for the reduced number of search movements by increasing the distance between individual eye movements; rather, the exploratory behavior itself was likely constrained.

Regarding sign location, only the number of saccades decreased in the left condition, whereas the fixation duration did not differ from that in the front condition. Thus, left-side guide sign placement may not reduce the time spent “looking at” the destination itself, but instead constitutes a condition in which exploratory eye movements between the forward environment and sign are more constrained [57]. That is, rapid gaze switching is constrained for left-sided signs, potentially rendering information integration through exploration more challenging.

Overall, both increased driving speed and left-side sign placement reduced the temporal and attentional leeway available for visual search, creating conditions in which exploratory eye movements are more readily suppressed. Under such conditions, reliance on automated search strategies based on driving experience increases, whereas under less demanding conditions, individual differences in cognitive function are more likely to be reflected in eye-movement behavior.

### 4.4. Limitations of the Study

This study has some limitations. First, standardized on-board videos were employed as stimuli. Although viewing conditions were controlled across participants through the use of identical videos, the stimuli inevitably differed from those in driving simulators in which drivers can actively control the vehicle’s speed and position [62]. This difference may have influenced eye movements. Therefore, this study should be regarded as a preliminary investigation, such as using driving simulators or actual on-road experiments in which participants operate a vehicle and recognize specific guide signs in real time. These approaches would enable the examination of cognitive processing and gaze behavior under more ecologically valid conditions. Future studies should be conducted under conditions that more closely approximate real-world driving conditions.

Second, although the sample size for this study was determined using G*Power, it may have been insufficient to reliably detect small-to-moderate effect sizes. Nevertheless, the primary focus of this study was validating large effect sizes, which were particularly significant. Specifically, large effect sizes clearly highlighted issues related to visibility and cognitive load in real-world driving environments, thereby providing practical guidance for optimizing guide sign placement and advancing driver assistance technologies. In addition, the present sample included more women than men (16 women and 8 men). Although this gender imbalance is unlikely to have affected the main findings, it may limit the generalizability of the results to a broader driving population. Future research should undertake more comprehensive investigations across a broader spectrum of effect sizes. Replication studies with larger sample sizes are warranted to enhance the reproducibility and generalizability of the findings.

Third, this study measured two types of eye movement: fixations and saccades. However, smooth-pursuit eye movements have also been documented when the eyes track moving objects, including text [63]. The velocity of this type of eye movement is approximately 30°/s in terms of visual angle, which is slower than that of saccades [64]. Thus, the eye tracker used in this study (Tobii X60, 60 Hz) was unable to record smooth-pursuit eye movements with sufficient temporal resolution. Smooth-pursuit movements have been demonstrated to be relevant in the context of driving [64,65]. Therefore, future research using eye-tracking systems with higher temporal resolution should investigate the role of smooth-pursuit eye movements in destination recognition during driving. Furthermore, performance verification in this study relied solely on a binary self-report (“recognized the destination”). This subjective measure did not include objective behavioral data such as response accuracy or reaction time, which could provide more detailed insights into recognition performance. Therefore, future studies should complement subjective self-reports with objective behavioral measures, including reaction time and response accuracy. These approaches allow for a more precise and multifaceted evaluation of destination recognition processes in guide signs.

Finally, to approximate real-world driving scenarios, we prioritized reproducing the natural variation in sign approach corresponding to vehicle speed, while keeping the visual recognition time of the signs constant. However, this approach imposes limitations on internal validity, as the initial viewing distance and the timing at which signs become visible were not standardized across speed conditions. Moreover, the order of stimulus presentation was fixed (slow → fast), rather than randomized or counterbalanced, which may have introduced learning or fatigue effects. Although the GLMM framework allowed us to statistically control for participant- and video-level variability, slow- and fast-speed videos were recorded on different road types (general roads and highways), and the potential confounding factors inherent to these environments (e.g., sign size, visual clutter, and landscape features) could not be completely eliminated. Future studies should more rigorously control these factors by designing conditions that unify the presentation distance and onset timing of signs or by adjusting the visible duration and condition for each sign individually to better isolate their effects.

## 5. Conclusions

Eye-movement behavior during guide sign recognition while driving was shaped by the interaction between driving conditions and driving speed (slow vs. fast) and sign location (front vs. left)—and drivers’ cognitive functions and individual characteristics. Under low-demand conditions, such as slow driving and front sign placement, individual differences in cognitive functions, including verbal memory and UFOV, were more readily reflected in eye-movement behaviors. By contrast, under high-demand conditions, such as fast driving and left-side sign placement, factors based on driving experience and strategic adaptation govern eye-movement behavior more strongly. As task demands increase, reliance may shift from flexible cognitive processing to automated search strategies grounded in experience.

Furthermore, an increased driving speed consistently reduced the number of fixations and saccades, indicating a general suppression of exploratory eye movements. By contrast, under left-side sign placement, fixation duration was maintained, while the number of saccades decreased, suggesting constraints on exploratory gaze shifts between the forward environment and the sign. Thus, left-side guide signs may impose a qualitatively higher level of demand, not by shortening viewing time but by making gaze allocation and information integration more difficult.

Overall, guide sign recognition has emerged as a process driven by the interaction between environmental and individual factors. In high-demand situations such as fast driving and left-sided sign placement, the influence of cognitive functions may be relatively attenuated, with greater reliance on experience and search strategies. The findings of this study provide foundational insights for human-centered traffic infrastructure design, including guide sign placement and information load optimization, and for the development of driver support strategies.

## Figures and Tables

**Table 1:** Details of the participants’ cognitive functions.

Assessment Measures	Score (Mean ± Standard Deviation, (Range)
TMT-J A (seconds)	33.89 ± 10.97 (22.61–69.15)
TMT-J B (seconds)	63.16 ± 44.15 (29.1–260)
WMS-R verbal memory	22.33 ± 5.23 (10–30)
WMS-R visual memory	36.83 ± 4.16 (27–41)
BADS Zoo Map Test	15.58 ± 0.64 (14–16)
UFOV	50 ± 2.73 (43–56)

**Table 2:** Interactions between driving speed and basic demographics as well as cognitive functions.

Eye Movement Measure	Interaction	β	95% CI	F(df1, df2)	*p*	η^2^
**Fixation duration**	Speed × age	−0.146	[−1.958, 1.665]	F(1, 950) = 0.025	0.874	<0.001
	Speed × driving history	−0.384	[−2.000, 1.232]	F(1, 950) = 0.217	0.641	<0.001
	Speed × educational history	6.937	[−5.648, 19.522]	F(1, 950) = 1.170	0.280	0.001
	Speed × TMT-J A	−0.144	[−2.320, 2.033]	F(1, 950) = 0.017	0.897	<0.001
	Speed × TMT-J B	−0.378	[−0.919, 0.162]	F(1, 950) = 1.890	0.170	0.002
	Speed × WMS-R verbal memory	−4.192	[−8.761, 0.377]	F(1, 950) = 3.242	0.072	0.003
	Speed × WMS-R visual memory	−0.577	[−6.233, 5.079]	F(1, 950) = 0.040	0.841	<0.001
	Speed × ZMT	−23.623	[−60.864, 13.618]	F(1, 950) = 1.550	0.214	0.002
	Speed × UFOV	−4.353	[−13.041, 4.335]	F(1, 950) = 0.967	0.326	0.001
**Number of fixations**	Speed × age	−0.006	[−0.010, −0.001]	F(1, 950) = 5.266	0.022 *	0.006
	Speed × driving history	−0.004	[−0.009, −0.000]	F(1, 950) = 4.067	0.037 *	0.004
	Speed × educational history	−0.020	[−0.052, 0.012]	F(1, 950) = 1.498	0.221	0.002
	Speed × TMT-J A	−0.001	[−0.006, 0.005]	F(1, 950) = 0.043	0.836	<0.001
	Speed × TMT-J B	−0.000	[−0.001, 0.001]	F(1, 950) = 0.004	0.947	<0.001
	Speed × WMS-R verbal memory	0.012	[0.001, 0.024]	F(1, 950) = 4.386	0.037 *	0.005
	Speed × WMS-R visual memory	−0.002	[−0.016, 0.013]	F(1, 950) = 0.056	0.813	<0.001
	Speed × ZMT	−0.079	[−0.172, 0.015]	F(1, 950) = 2.732	0.099	0.003
	Speed × UFOV	0.024	[0.002, 0.046]	F(1, 950) = 4.432	0.036 *	0.005
**Saccade amplitude**	Speed × age	0.004	[−0.025, 0.032]	F(1, 953) = 0.067	0.796	<0.001
	Speed × driving history	0.003	[−0.023, 0.028]	F(1, 953) = 0.037	0.847	<0.001
	Speed × educational history	−0.101	[−0.294, 0.092]	F(1, 954) = 1.048	0.306	0.001
	Speed × TMT-J A	−0.000	[−0.034, 0.033]	F(1, 953) = 0.000	0.996	<0.001
	Speed × TMT-J B	−0.002	[−0.011, 0.006]	F(1, 953) = 0.298	0.585	<0.001
	Speed × WMS-R verbal memory	0.008	[−0.063, 0.078]	F(1, 953) = 0.047	0.829	<0.001
	Speed × WMS-R visual memory	−0.051	[−0.139, 0.038]	F(1, 953) = 1.268	0.261	0.001
	Speed × ZMT	0.139	[−0.435, 0.713]	F(1, 953) = 0.226	0.635	<0.001
	Speed × UFOV	0.053	[−0.082, 0.188]	F(1, 953) = 0.596	0.440	0.001
**Number of saccades**	Speed × age	−0.003	[−0.008, 0.002]	F(1, 950) = 1.769	0.184	0.002
	Speed × driving history	−0.003	[−0.007, 0.002]	F(1, 950) = 1.457	0.228	0.002
	Speed × educational history	−0.021	[−0.052, 0.011]	F(1, 950) = 1.692	0.194	0.002
	Speed × TMT-J A	0.000	[−0.005, 0.006]	F(1, 950) = 0.001	0.973	<0.001
	Speed × TMT-J B	−0.000	[−0.001, 0.001]	F(1, 950) = 0.001	0.977	<0.001
	Speed × WMS-R verbal memory	0.015	[0.003, 0.026]	F(1, 950) = 6.567	0.011 *	0.007
	Speed × WMS-R visual memory	0.002	[−0.013, 0.016]	F(1, 950) = 0.046	0.830	<0.001
	Speed × ZMT	−0.094	[−0.186, −0.002]	F(1, 950) = 4.036	0.045 *	0.004
	Speed × UFOV	0.019	[−0.003, 0.041]	F(1, 950) = 2.939	0.087	0.003

Note: Cells with *p*-values marked with asterisks and shaded in gray indicate combinations for which statistically significant effects were observed. ZMT, Zoo Map Test; TMT, Trail Making Test; CI, confidence interval; UFOV, useful field of view; WMS-R, Wechsler Memory Scale—Revised.

**Table 3:** Interactions between sign location and basic demographics as well as cognitive functions.

Eye Movement Measure	Interaction	β	95% CI	F(df1, df2)	*p*	η^2^
**Fixation duration**	Location × age	0.740	[−1.070, 2.551]	F(1, 950) = 0.644	0.423	<0.001
	Location × driving history	0.709	[−0.906, 2.325]	F(1, 950) = 0.743	0.389	<0.001
	Location × educational history	2.368	[−10.224, 14.960]	F(1, 950) = 0.136	0.712	<0.001
	Location × TMT-J A	−1.034	[−3.209, 1.142]	F(1, 950) = 0.869	0.351	<0.001
	Location × TMT-J B	−0.102	[−0.643, 0.439]	F(1, 950) = 0.138	0.711	<0.001
	Location × WMS-R verbal memory	−3.778	[−8.348, 0.792]	F(1, 950) = 2.631	0.105	0.003
	Location × WMS-R visual memory	−4.498	[−10.147, 1.151]	F(1, 950) = 2.442	0.119	0.003
	Location × BADS Zoo Map	20.405	[−16.844, 57.654]	F(1, 950) = 1.156	0.283	0.001
	Location × UFOV	−5.106	[−13.792, 3.580]	F(1, 950) = 1.331	0.249	0.001
**Number of fixations**	Location × age	−0.003	[−0.007, 0.002]	F(1, 950) = 1.207	0.272	0.001
	Location × driving history	−0.002	[−0.007, 0.002]	F(1, 950) = 1.225	0.269	0.001
	Location × educational history	−0.034	[−0.066, −0.003]	F(1, 950) = 4.481	0.035 *	0.005
	Location × TMT-J A	−0.003	[−0.009, 0.002]	F(1, 950) = 1.398	0.237	0.001
	Location × TMT-J B	−0.000	[−0.001, 0.001]	F(1, 950) = 0.011	0.916	<0.001
	Location × WMS-R verbal memory	−0.008	[−0.019, 0.004]	F(1, 950) = 1.722	0.190	0.002
	Location × WMS-R visual memory	−0.007	[−0.022, 0.008]	F(1, 950) = 0.884	0.347	<0.001
	Location × BADS Zoo Map	0.050	[−0.044, 0.144]	F(1, 950) = 1.095	0.296	0.001
	Location × UFOV	−0.004	[−0.027, 0.018]	F(1, 950) = 0.147	0.701	<0.001
**Saccade amplitude**	Location × age	−0.002	[−0.031, 0.027]	F(1, 953) = 0.016	0.899	<0.001
	Location × driving history	−0.001	[−0.026, 0.025]	F(1, 953) = 0.005	0.945	<0.001
	Location × educational history	0.092	[−0.101, 0.285]	F(1, 954) = 0.869	0.351	<0.001
	Location × TMT-J A	0.004	[−0.029, 0.038]	F(1, 953) = 0.065	0.799	<0.001
	Location × TMT-J B	0.003	[−0.005, 0.011]	F(1, 953) = 0.541	0.462	<0.001
	Location × WMS-R verbal memory	−0.021	[−0.092, 0.049]	F(1, 953) = 0.357	0.550	<0.001
	Location × WMS-R visual memory	0.064	[−0.025, 0.152]	F(1, 953) = 2.003	0.157	0.002
	Location × BADS Zoo Map	−0.114	[−0.688, 0.461]	F(1, 953) = 0.151	0.698	<0.001
	Location × UFOV	−0.071	[−0.206, 0.064]	F(1, 953) = 1.074	0.300	0.001
**Number of saccades**	Location × age	−0.004	[−0.008, 0.001]	F(1, 950) = 2.344	0.126	0.002
	Location × driving history	−0.003	[−0.007, 0.001]	F(1, 950) = 2.181	0.140	0.002
	Location × educational history	−0.030	[−0.061, 0.002]	F(1, 950) = 3.468	0.063	0.004
	Location × TMT-J A	−0.003	[−0.008, 0.002]	F(1, 950) = 1.139	0.286	0.001
	Location × TMT-J B	0.000	[−0.001, 0.002]	F(1, 950) = 0.079	0.779	<0.001
	Location × WMS-R verbal memory	−0.013	[−0.025, −0.002]	F(1, 950) = 5.302	0.022 *	0.006
	Location × WMS-R visual memory	−0.008	[−0.023, 0.006]	F(1, 950) = 1.338	0.248	0.001
	Location × BADS Zoo Map	0.062	[−0.030, 0.154]	F(1, 950) = 1.740	0.188	0.002
	Location × UFOV	−0.009	[−0.031, 0.013]	F(1, 950) = 0.688	0.407	<0.001

Note: Cells with *p*-values marked with asterisks and shaded in gray indicate combinations for which statistically significant effects were observed. ZMT, Zoo Map Test; TMT, Trail Making Test; CI, confidence interval; UFOV, useful field of view; WMS-R, Wechsler Memory Scale—Revised.

**Table 4:** Main effects of driving speed and sign location on eye movement measures.

Eye Movement Measures	Factor	β	95% CI	F(df1, df2)	*p*	SE
**Fixation duration**	Speed	−38.253	[−62.169, −14.338]	F(1, 950) = 9.854	0.002	12.186
	Location	−16.480	[−40.395, 7.435]	F(1, 950) = 1.829	0.177	12.186
**Number of fixations**	Speed	−0.226	[−0.287, −0.165]	F(1, 950) = 53.348	<0.001	0.031
	Location	0.018	[−0.043, 0.079]	F(1, 950) = 0.334	0.563	0.031
**Saccade amplitude**	Speed	−0.312	[−0.680, 0.056]	F(1, 956) = 2.771	0.096	0.187
	Location	−0.185	[−0.553, 0.183]	F(1, 956) = 0.978	0.323	0.187
**Number of saccades**	Speed	−0.182	[−0.242, −0.122]	F(1, 950) = 35.276	<0.001	0.031
	Location	0.065	[0.005, 0.125]	F(1, 950) = 4.508	0.034	0.031

CI, confidence interval; SE, standard error.

## Data Availability

The datasets generated and analyzed during this study are not publicly available due to the inclusion of personally identifiable information. However, they may be made available from Hiroki Okada (e-mail: h-okada@pop.med.hokudai.ac.jp) upon reasonable request and subject to ethical approval.

## Supplementary Materials

The following supporting information can be downloaded at: https://www.mdpi.com/article/10.3390/jemr19020025/s1, Table S1. Correlations among cognitive functions and participants’ basic demographic variables (r/P); Figure S1. Lower-triangle correlation matrix of cognitive functions and participants’ basic demographic variables (significant correlations marked with *).
