# Supplementary material for: Characteristics of Eye Movements and Correlation to Cognitive Functions in Relation to the Location of Guide Signs and Driving Speed
Source: J Eye Mov Res. 2026 Mar 2;19(2):25. doi: 10.3390/jemr19020025 (PMC13010679; doi:10.3390/jemr19020025)
Supplement: Supplementary file 1 [file jemr-19-00025-s001.zip › Supplementary Figure S1.pdf]

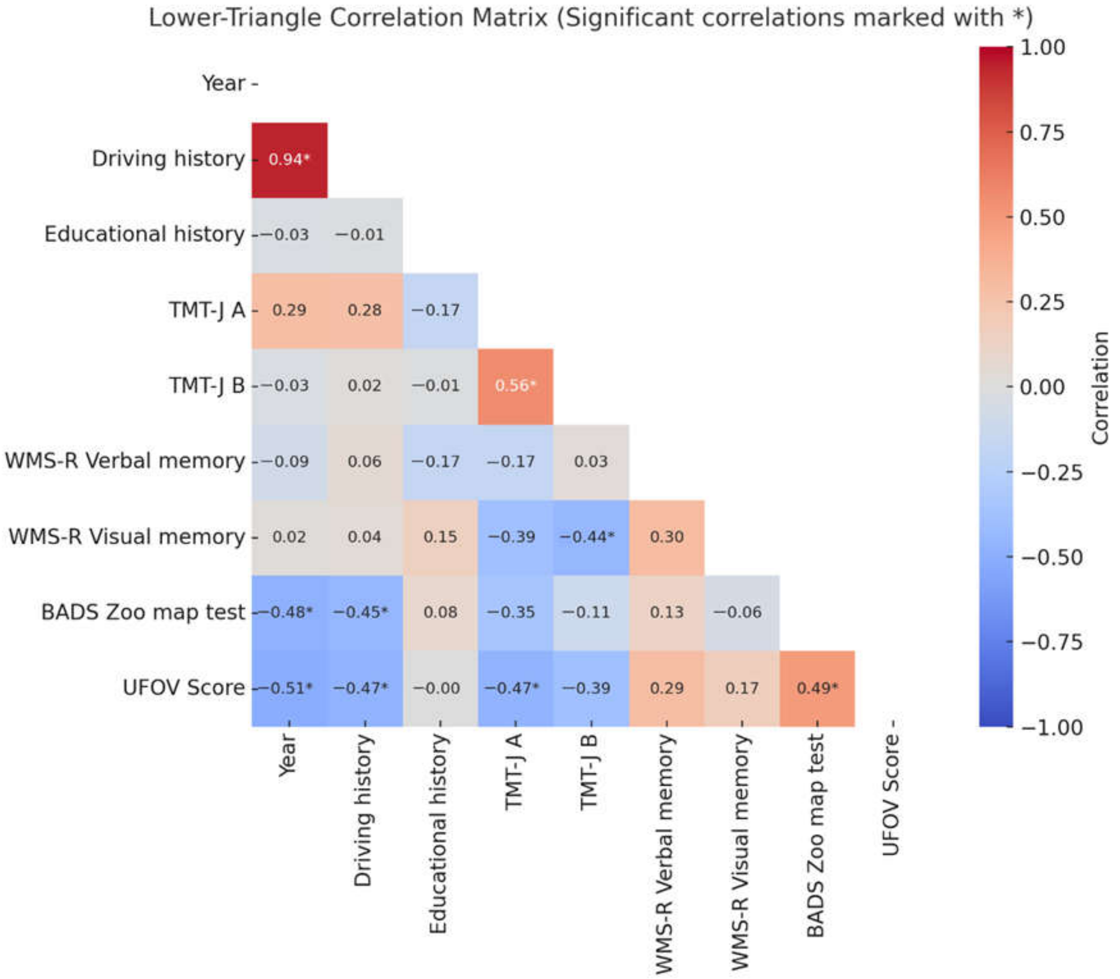

Figure S1. Lower-triangle correlation matrix of cognitive functions and participants' basic demographic variables (significant correlations marked with *)
