# Supplementary material for: Characteristics of Eye Movements and Correlation to Cognitive Functions in Relation to the Location of Guide Signs and Driving Speed
Source: J Eye Mov Res. 2026 Mar 2;19(2):25. doi: 10.3390/jemr19020025 (PMC13010679; doi:10.3390/jemr19020025)
Supplement: Supplementary file 1 [file jemr-19-00025-s001.zip › Supplementary Table S1.pdf]

**Table S1.** Correlations among cognitive functions and participants' basic demographic variables (r/P)

| Variables | Year | Driving history | Educational history | TMT-J A | TMT-J B | WMS-R Verbal memory | WMS-R Visual memory | BADS Zoo Map Test | UFOV Score |
|---|---|---|---|---|---|---|---|---|---|
| **Year** | — | | | | | | | | |
| **Driving history** | **0.94 / < 0.01** | — | | | | | | | |
| **Educational history** | −0.03 / 0.87 | −0.01 / 0.96 | — | | | | | | |
| **TMT-J A** | 0.29 / 0.17 | 0.28 / 0.19 | −0.17 / 0.42 | — | | | | | |
| **TMT-J B** | −0.03 / 0.89 | 0.02 / 0.94 | −0.01 / 0.95 | **0.56 / < 0.01** | — | | | | |
| **WMS-R Verbal memory** | −0.09 / 0.69 | 0.06 / 0.77 | −0.17 / 0.43 | −0.17 / 0.43 | 0.03 / 0.87 | — | | | |
| **WMS-R Visual memory** | 0.02 / 0.93 | 0.04 / 0.85 | 0.15 / 0.47 | −0.39 / 0.06 | −0.44 / 0.03 | 0.30 / 0.16 | — | | |
| **BADS Zoo Map test** | **−0.48 / 0.02** | **−0.45 / 0.03** | 0.08 / 0.70 | −0.35 / 0.09 | −0.11 / 0.62 | 0.13 / 0.55 | −0.06 / 0.79 | — | |
| **UFOV Score** | **−0.51 / 0.01** | **−0.47 / 0.02** | −0.00 / 1.00 | **−0.47 / 0.02** | −0.39 / 0.06 | 0.29 / 0.17 | 0.17 / 0.42 | **0.49 / 0.01** | — |

NOTE: Boldface indicates combinations with P < 0.05. To avoid redundancy, only the lower triangle is displayed.
